# Prodromal Symptoms of Acute Myocardial Infarction in Women: A Systematic Review of Current Evidence

**DOI:** 10.1002/nop2.70211

**Published:** 2025-04-22

**Authors:** Vincenza Giordano, Rita Nocerino, Caterina Mercuri, Teresa Rea, Assunta Guillari

**Affiliations:** ^1^ Department of Biomedicine and Prevention University of Rome Tor Vergata Rome Italy; ^2^ Department of Translational Medical Science Federico II University Hospital Naples Italy; ^3^ ImmunoNutritionLab, CEINGE‐Advanced Biotechnologies University of Naples “Federico II” Naples Italy; ^4^ Department of Experimental and Clinical Medicine Magna Graecia University of Catanzaro Catanzaro Italy; ^5^ Public Health Department Federico II University Hospital Naples Italy

**Keywords:** acute coronary syndrome, acute myocardial infarction, prodromal symptom, woman

## Abstract

**Aim:**

To synthezise quantitative current evidence on the prodromal symptoms experienced by women before the onset of acute coronary syndrome (ACS), focusing on the prevalence, nature and clinical implications of these symptoms.

**Design:**

A systematic review.

**Methods:**

The review adhered to Synthesis without meta‐analysis guidelines and was registered with the PROSPERO database (ID: CRD42024541840). Systematic searches were conducted in PubMed, CINAHL, APA PsycArticles, APA PsycInfo and EMBASE. Included studies were quantitative, focused on women aged ≥ 18 years with confirmed acute coronary syndrome and reported prodromal symptoms. The Quality Assessment with Diverse Studies and the Cochrane Risk of Bias in non‐randomized studies of Interventions tools were used for critical appraisal.

**Results:**

Of 2170 identified records, 11 full‐text studies were reviewed. The most frequently reported prodromal symptom was unusual fatigue, followed by sleep disturbances and anxiety. Prodromal symptoms often occurred well before the acute event but were frequently misattributed to non‐cardiac causes. Chest pain, typically associated with acute coronary syndrome, was less commonly reported as a prodromal symptom in women, complicating timely diagnosis and treatment.

**Conclusions:**

The results highlight the need for increased awareness of these early warning signs among healthcare providers and women themselves. Enhanced recognition and understanding of these symptoms could lead to more timely and accurate diagnosis, ultimately improving outcomes for women at risk of acute myocardial infarction.

**Implications for the Profession and/or Patient Care:**

To educate both health professionals and patients about the variability and significance of prodromal symptoms in women is essential to improve outcomes.

**Impact:**

This study is the first to systematically review and synthesize the existing literature on prodromal symptoms of acute coronary syndrome specifically in women. The results show l that women are more likely to experience a broader and more complex range of prodromal symptoms, including fatigue, sleep disturbances and anticipatory anxiety, which often precede the acute event. The insights provided by our review could lead to significant improvements in the early diagnosis and treatment of AMI in women, ultimately reducing morbidity and mortality rates associated with cardiovascular diseases.

**Reporting Method:**

The review has adhered to relevant EQUATOR guidelines and has followed Synthesis without meta‐analysis guidelines.

**Patient or Public Contribution:**

No patient or public contribution.

## 1 Introduction

Cardiovascular disease (CVD) remains a leading cause of morbidity and mortality among women, yet it is often underdiagnosed, undertreated and under‐researched, particularly concerning the prevention, diagnosis and treatment of heart disease (Vogel et al. 2021). Among the various forms of CVD, ischaemic heart disease is the most prevalent and deadly, with an estimated 197 million cases and 9.14 million deaths globally (Rodriguez Lozano et al. 2022). While men account for 114 million cases, women represent 83.6 million cases, with a mortality rate of 4.17 million, highlighting a significant gender disparity in both prevalence and outcomes (Roth et al. 2020). In Europe, ischaemic heart disease accounts for 40% of cardiovascular deaths in women, underscoring the critical need for targeted interventions (Townsend et al. 2022).

Ischaemic heart disease includes acute myocardial infarction (AMI) with non‐obsctructive coronary artery disease (MINOCA) and non‐obstructive ischaemia and coronary artery disease (INOCA) (Vogel et al. 2021; Pacheco et al. 2022). A significant subset of ischaemic heart disease is represented by acute coronary syndrome (ACS), a term used to describe a group of conditions caused by sudden, reduced blood flow to the heart. ACS encompasses ST‐elevation myocardial infarction (STEMI), non‐ST‐elevation myocardial infarction (NSTEMI) and unstable angina, which are distinguished by their severity and clinical presentation. ACS is often caused by the rupture of an atherosclerotic plaque, resulting in partial or complete obstruction of coronary blood flow (Singh et al. 2023).

Women with AMI often experience intermittent symptoms that may last for hours, days or weeks before the acute event. Although chest pain is the most common symptom, women often present more heterogeneous symptoms of AMI (Cardeillac et al. 2022). Indeed, symptoms such as breathing difficulties, digestive problems and fatigue have traditionally been defined as ‘atypical’. However, this classification of ‘atypicality’ has been questioned over the years, as it is increasingly believed that the variability of symptoms is influenced by gender (DeVon et al. 2020; Cardeillac et al. 2022). In addition, the variability and intermittency of these symptoms may lead to misinterpretation of AMI by both patients and healthcare professionals, hindering early recognition and often leading to misdiagnosis (Lichtman et al. 2015; Birnbach et al. 2020).

The focus on prodromal symptoms of AMI dates to the 1930s (Feil 1937). Prodromal symptoms are generally considered warning signs of an impending ACS. They represent the early stages of heart disease and tend to be short‐lived, less intense, transient and non‐specific compared to unstable angina (O'Keefe‐McCarthy and Ready 2016). These symptoms lack specific features, are transient, indicative of an impending ACS and may occur days to several weeks before an adverse cardiac event (O'Keefe‐McCarthy and Ready 2016). Initially, in fact, studies such as Nixon and Bethell (1974) underscored the importance of symptoms like fatigue and pain as early indicators of AMI, yet they did not account for gender differences; later research, then, has explored this aspect more thoroughly, demonstrating a broader spectrum of prodromal symptoms in women. For example, Hofgren et al. (1995) study found that 70% of women reported symptom onset before hospital admission. More specifically, a study by McSweeney and Crane (2000) involving 40 women with AMI revealed that 92.5% reported at least one prodromal symptom, with 87.5% experiencing three or more symptoms, which manifested 4–6 months prior to the event.

According to Rosenfeld (2001), the severity of symptoms and their slow or intermittent progression are crucial clinical factors predicting delays in seeking medical attention among women; Kirchberger et al. (2011) report that the female sex is associated with a higher risk of misinterpreting the symptoms of AMI: women not only fail to exhibit the typical symptoms of AMI (Kirchberger et al. 2011), but often do not perceive themselves to be at risk for AMI (Lichtman et al. 2015). In addition, they tend not to associate their symptoms with a cardiac problem, but rather with anxiety and stress, and react to the event in different ways (Buckley et al. 2007; Kirchberger et al. 2011). Not only women but also healthcare professionals show delays and inconsistencies in responding to prodromal symptoms (Lichtman et al. 2015). Such behaviors can lead to delays in seeking care, with potential negative consequences for the effectiveness of treatment or on the timing of interventions (Kirchberger et al. 2011; Lichtman et al. 2015). Bahr et al. (2001) emphasized that early recognition of prodromal symptoms of AMI is a critical but often neglected and underestimated issue that could benefit from targeted educational interventions for both patients and healthcare providers. To date, there have been no systematic reviews of the quantitative evidence, but there has been an integrative review by Blakeman and Booker (2016).

Existing reviews in the literature focus on prodromal symptoms across both sexes, investigate a single prodromal symptom in women, assess prodromal and acute symptoms together or explore these symptoms in relation to risk factors. For instance, Miller's (2002) review explored qualitative and quantitative studies on cardiac warning symptoms in women, while Blakeman and Stapleton (2018) conducted an integrative review addressing the severity, discomfort, quality and timing of prodromal AMI fatigue in women, offering recommendations for clinical practice and future research. In contrast, Schulte and Mayrovitz (2023) conducted a systematic review analysing differences in symptoms and pathophysiology of AMI between women and men, and Bruyninckx et al. (2008) focused on the accuracy of 10 signs and symptoms in both genders with AMI and ACS.

Therefore, a systematic review is urgently needed to consolidate the current evidence and improve our understanding of the unique prodromal symptomatology of ACS in women, thereby improving diagnostic accuracy and patient outcomes.

## 2 Aim of Study

The aim of this systematic review is to synthesize and critically analyze the existing quantitative evidence on the prodromal symptoms experienced by women before to the onset of ACS.

## 3 Design and Methods

### 3.1 Design

This systematic review was conducted following the Synthesis without meta‐analysis (SWiM) (Campbell et al. 2020). This structured approach enabled a comprehensive and methodical analysis of literature. It focused on identifying, selecting, evaluating and synthesising data related to prodromal symptoms of AMI in women. The protocol of the systematic review was registered in the PROSPERO database. The registration number was CRD42024541840.

### 3.2 Data Sources and Search Strategies

This systematic review was conducted using a rigorous, evidence‐based approach. We hypothezised that women experience prodromal symptoms in the days before an ACS. The research question for this systematic review was ‘*What are the most common prodromal symptoms reported by women diagnosed with acute coronary syndrome*?’ The population, intervention, outcome (PIO) framework was utilized to identify keywords and MeSH terms that included: the target population (women with ACS), interventions (symptom recognition) and outcomes (prodromal symptoms). Relevant publications were identified by systematic data searches from March to June 2024 in the following electronic databases: Pubmed, CINAHL, APA PsycArticles, APA PsycInfo, EMBASE. Combinations of the following medical key words were used: “woman,” “female,” “prodromal symptoms,” “early warning,” “premonitory symptoms,” “acute coronary syndrome,” “acute myocardial infarction,” “heart attack,” “ischemic heart disease” and “acute disease.” In each database, synonyms were identified for each Medical Subject Heading (MeSH) term. Advanced search strings were created using Boolean operators such as OR and AND to cross‐reference these terms in various combinations to increase the specificity of the search (Supporting Information).

It is important to note that no filters were applied to the database searches. The formulation of keywords and MeSH terms was a collaborative effort involving all authors to meet validity criteria and reduce search bias. Similary, the authors collaborated in the retrieval of studies and full texts and jointly assesed them to identify reports relevant to the research topic. The research team reviewed the titles and the abstracts. No date restrictions were applied when searching for articles in the databases.

### 3.3 Selection Criteria

To be eligible for inclusion, studies were subjected to the following inclusion criteria:

- Primary quantitative studies;
- Studies in adult female populations (≥ 18 years) with a confirmed diagnosis of acute coronary syndrome (ACS), including ST‐segment elevation myocardial infarction (STEMI), non‐ST‐segment elevation myocardial infarction (NSTEMI) or unstable angina, who presented prodromal symptoms;
- Studies that numerically described one or more specific prodromal symptoms of myocardial infarction or a group of symptoms experienced by women;
- English‐language studies only.

The studies were excluded based on the following criteria:

- Secondary and qualitative studies;
- Editorial reviews and/or letters to the editor;
- Dissertations;
- Studies in which there is complexity in sampling adult women (≥ 18 years) with a confirmed diagnosis of acute myocardial infarction who have prodromal symptoms;
- Studies in which it is difficult to identify or extract prodromal symptoms (e.g., studies that examine both prodromal and acute symptoms);
- Studies that did not report a specific focus on prodromal symptoms;
- Studies in languages other than English.

In this review, no temporal limits were applied to the presentation of prodromal symptoms of the acute event.

### 3.4 Study Screening and Data Extraction

The selected studies were uploaded to the Rayyan platform (https://www.rayyan.ai) for preliminary screening.

**FIGURE 1:**
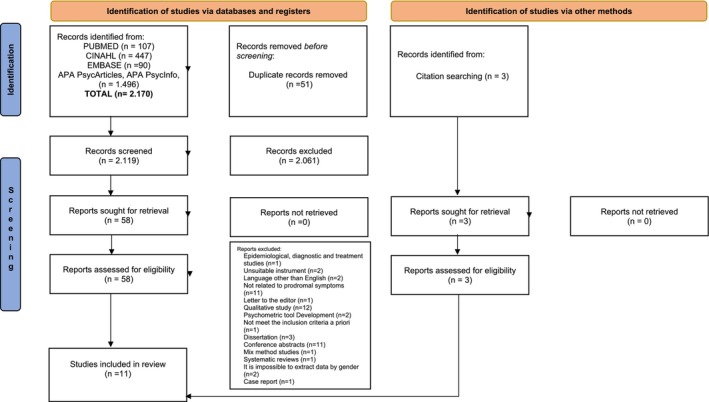
PRISMA flow diagram.

Three reviewers (V.G., C.M., A.G.) performed the screening and selection of these studies. After duplicates were eliminated, three investigators (V.G., C.M., A.G.) independently assessed study titles and abstracts from five databases to exclude irrelevant studies based on the eligibility criteria. In the next step, the researchers assessed the remaining study types and accessed the full texts of potentially relevant studies. Discrepancies in study inclusion were resolved by discussion with a fourth reviewer (R.N.). From the final 11 studies, data were extracted and independently reviewed by three researchers (V.G., C.M., A.G.). The eligibility of the studies for inclusion in the review was determined by consensus of all authors. The screening process is illustrated in the PRISMA diagram (Figure 1) (Page et al. 2021).

### 3.5 Data Synthesis and Evaluation

After the identification of the literature to be included in the review, the studies were read once to gain a general understanding of the body of literature . Three authors (V.G., C.M., A.G.) independently extracted data, including the author, year of publication, study purpose, sample size and demographics, outcome measures, instruments or scales used, study methodology, statistical analyses, results relevant to the research question and including any limitations noted.

Several critical factors made a meta‐analysis impossible, as it would have compromised the methodological integrity. Significant heterogeneity was observed among the studies in terms of research designs and study populations. Differences in sample sizes and methodologies limited the generalizability of the results and posed challenges in pooling data for a meta‐analysis. Variability in the scales and metrics used to assess outcomes further complicated the analysis.

Given these considerations, the review team concluded that a meta‐analysis was not feasible due to the heterogeneity of the data and therefore opted for a narrative synthesis to provide a comprehensive overview of prodromal symptoms in women prior to the diagnosis of AMI. This approach allows for a qualitative exploration of literature, acknowledging its complexity and variation, yet providing valuable insights. Due to the heterogeneity among the examined studies, the studies were grouped by types of prodromal symptoms reported by women: anxiety, fatigue, sleep disturbance, changesperformed in thinking and memory, dyspnea and respiratory problems, gastrointestinal symptoms, headache, other prodromal symptoms and pain.

### 3.6 Quality Assessment

The methodological quality of included studies was critically appraised in pairs using the Quality Assessment with Diverse Studies (QuADS) (Harrison et al. 2021) (Table 1). QuADS is an evolution of the quality assessment tool for studies with diverse designs (QATSDD) originally developed by Sirriyeh et al. (2012). The QuADS is an appraisal tool developed to determine the methodological and reporting quality of mixed‐ or multi‐method studies included in systematic reviews. The QuADS is designed to leverage survey data and literature review findings, thereby increasing its relevance to health services research, particularly for studies using mixed or multiple methods. Its widespread adoption underscores the value researchers place on the ability to assess the the quality of evidence from studies that use or combine different methods.

**TABLE 1:** Quality appraisal: Quality assessment with diverse studies (QuADS) (Harrison et al. 2021).

Study ID	Theoretical or conceptual research	Declaration of research objective(s)	Clear description of the setting and target population	The study design is appropriate to address the research objective(s)	Appropriate sampling to address the research objective(s)	Rationale for the choice of data collection instrument(s)	The format and content of the data collection tool is appropriate to address the stated research objective(s)	Description of the data collection procedure	Recruitment data provided	Motivation of the analytical method selected	The method of analysis was appropriate to meet the research objective(s)	Evidence that they were taken into account in the design or conduct of the research	Strengths and limitations critically discussed	Score
Cole et al. (2012)	0	3	3	3	1	3	3	0	0	0	3	0	3	22
Graham et al. (2008)	0	2	3	0	0	0	3	0	0	0	3	0	3	14
Joseph et al. (2021)	1	2	3	3	1	0	3	0	0	0	3	0	0	16
Khan et al. (2017).	0	1	3	3	0	0	3	0	0	0	3	0	3	16
Løvlien et al. (2006)	0	3	3	3	1	0	3	3	3	0	3	0	3	25
Løvlien et al. (2009)	1	3	3	3	2	2	3	3	3	0	3	0	3	29
McSweeney et al. (2003)	0	3	1	0	0	2	3	3	3	0	3	0	3	21
McSweeney et al. (2010)	0	3	3	3	3	0	3	3	3	0	3	0	3	27
O'Keefe‐McCarthy et al. (2016)	0	3	3	3	3	0	3	3	3	1	3	0	3	28
Shi et al. (2020)	0	2	3	3	3	0	3	1	0	0	3	0	2	20
Soltani et al. (2021)	0	3	2	3	1	0	3	3	0	0	3	0	0	18

The three authors (V.G., C.M., A.G.) independently assessed the methodological quality using the Cochrane Risk of Bias in Non‐Randomized Studies of Interventions (ROBINS‐I) tool (Sterne et al. 2016), which assesses the methodological quality of non‐randomized studies across seven bias domains (Table 2). This tool categorizes the risk of bias into four levels: ‘Critical’, ‘Serious’, ‘Moderate’ and ‘Low’, where a ‘Low’ risk corresponding to the risk of bias in a high‐quality randomized trial.

**TABLE 2:**
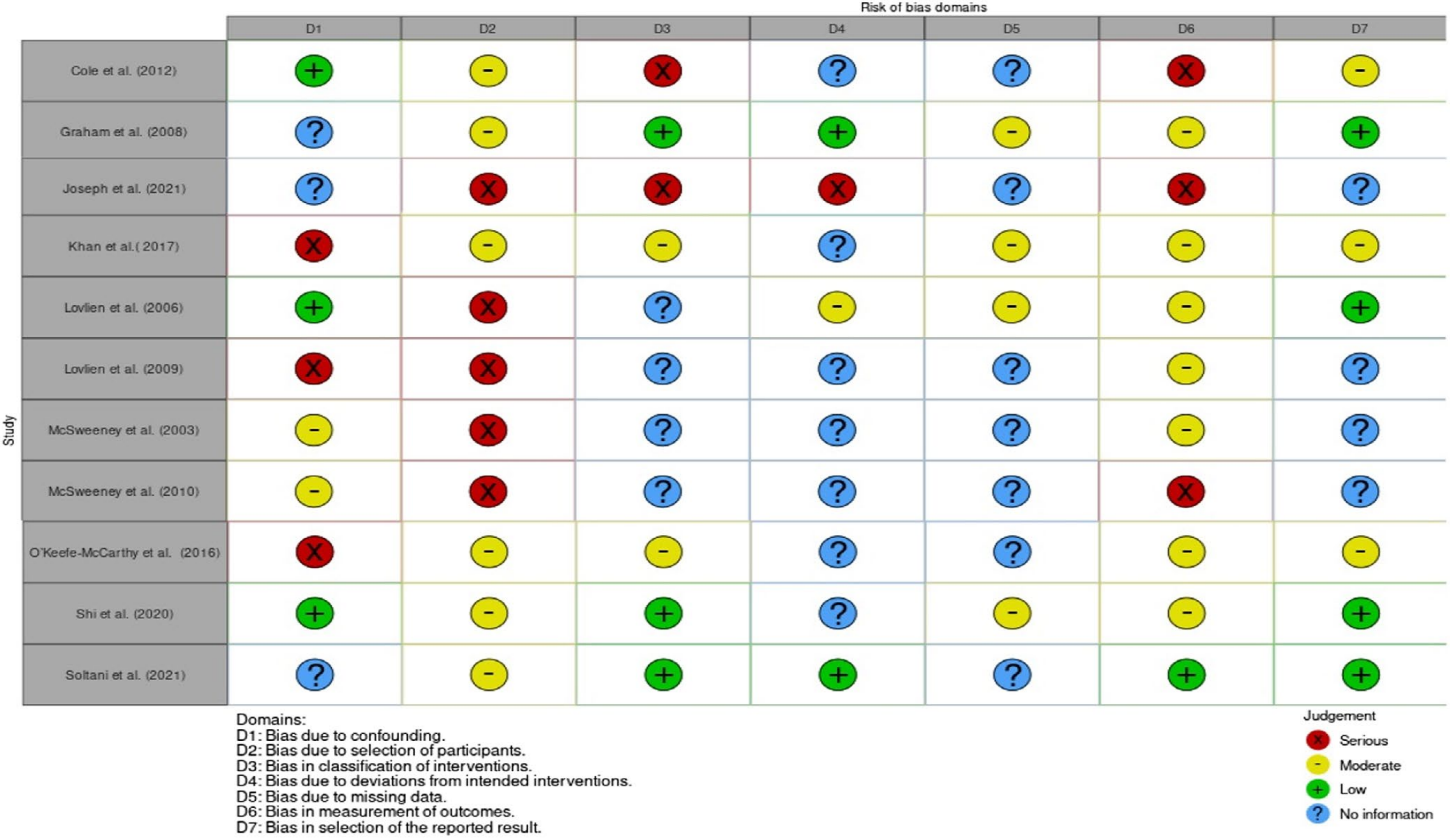
Risk of bias domains (ROBINS‐I) (Sterne et al. 2016).

## 4 Results

### 4.1 Study Selection

Our search strategy yielded a total of 2.170 studies in the initial stage, distributed as follows: 107 from PubMed, 90 from EMBASE, 447 from Cumulative Index to Nursing and Allied Health Literature (CINAHL) Complete, and 1.496 from APA PsycArticles, APA PsycInfo. 51 duplicate studies were excluded, leaving 2.119 for evaluation based on titles and abstracts. Of these, 2.061 were deemed irrelevant to the research objectives or inconsistent with the inclusion criteria. Full‐text access was obtained for all 58 remaining studies, which were then downloaded and thoroughly reviewed. Subsequently, 51 studies were excluded, while 8 articles met the criteria and were included in the review. Additionally, a supplementary free search was conducted, leading to the discovery of three articles. All full texts from this free search were downloaded. The systematic search yielded 11 studies published between 2003 and 2020. Figure 1 illustrates the PRISMA diagram (Page et al. 2021).

### 4.2 Study Characteristics

This systematic review included eight cross‐sectional studies (Cole et al. 2012; Løvlien et al. 2006, 2009; Khan et al. 2017; Shi et al. 2020; Soltani et al. 2021; Joseph et al. 2021; O'Keefe‐McCarthy et al. 2016), one retrospective study (McSweeney et al. 2010) and two descriptive studies (McSweeney et al. 2003; Graham et al. 2008). Nine studies have been conducted in developed countries, including Canada (Graham et al. 2008; O'Keefe‐McCarthy et al. 2016), China (Shi et al. 2020), Norway (Løvlien et al. 2006 and 2009), the United States (McSweeney et al. 2003, 2010; Cole et al. 2012) and one study was conducted across Canada, Switzerland and the United States (Khan et al. 2017). Two studies were instead conducted in two developing countries, Iran (Soltani et al. 2021) and India (Joseph et al. 2021).

The review includes a total of 5.543 women over of 18 years of age with ACS, who were studied for prodromal symptoms. Seven studies (McSweeney et al. 2003, 2010; Graham et al. 2008; Løvlien et al. 2009; O'Keefe‐McCarthy et al. 2016; Khan et al. 2017; Joseph et al. 2021) examined prodromal symptoms in both women and men but were included in the review as it was possible to extract data specifically for women, and the authors were precise and clear in analyzing the warning symptoms in both sexes.

There is considerable heterogeneity in the tools used to assess prodromal symptoms: most studies (Løvlien et al. 2006; McSweeney et al. 2003, 2013; Cole et al. 2012; Khan et al. 2017; Shi et al. 2020; Soltani et al. 2021) used the McSweeney Acute and Prodromal Myocardial Infarction Symptom Survey (MAPMISS) which includes descriptors for each symptom; women rate prodromal symptoms according to intensity (e.g., mild, severe) frequency (e.g., daily, weekly), and duration (e.g., 1 week or more than 1 month). MAPMISS also includes questions on comorbidity, risk factors, medications and demographic data (McSweeney et al. 2013); two studies (Løvlien et al. 2009; Joseph et al. 2021) used a specially designed self‐administered questionnaire; one study (O'Keefe‐McCarthy et al. 2016) used the Prodromal Symptoms‐Screening Scale (PS‐SS) which identifies nine cardiac‐related prodromal symptoms (unusually localized aches and/or pains, unusual fatigue, sleep disturbances, chest pain, anxiety, headache, dizziness and dyspnea) and finally one study (Graham et al. 2008) used the Ambulatory Care Classification System (ACCS) database, which contains up to 6 diagnostic queries, 10 procedure codes and the 5 potential groups of prodromal symptoms (pain, anxiety/fatigue, gastrointestinal disorders, head‐related disorders and others) (Graham et al. 2008).

Key characteristics of the studies were aggregated and synthesized by developing summary tables (Table 3). This approach provided an organized and clear synthesis of the main findings from the studies examined. The use of such graphical representations has thus facilitated the understanding and visualization of key information and provived an effective overview of the data generated by the literature review.

**TABLE 3:** Summarises the characteristics of included studies.

Author(s) & year	Title	Study objective	Instrument	Geographic region/nation	Study design	Results
Cole et al. (2012)	Sleep Disturbance in Women Prior to Myocardial Infarction	Describe the prevalence and correlations of sleep disturbances among women who retrospectively reported sleep disturbances before myocardial infarction (MI)	MAPMISS	United States	Cross‐sectional study *N* = 1270 women	Odds ratio (95% CI) Anxiety 2.21 (1.71, 2.84) Unusual fatigue 2.16 (1.62, 2.89) Change in thinking and memory 1.47 (1.12, 1.94) Headache 1.54 (1.03, 2.29) Gastrointestinal symptoms 1.29 (1.00, 1.67)
Graham et al. (2008)	Sex Differences in Patients Seeking Medical Attention for Prodromal Symptoms Before an Acute Coronary Event	Determine if there are sex differences in seeking medical attention for prodromal symptoms in patients who subsequently present to the emergency room with acute coronary syndrome (ACS)	Database ACS	Canada	Descriptive study *N* = 2268 (1234 women)	Anxiety and fatigue (52.4%) Headache (15.8%) Gastrointestinal symptoms (3.4%) Other symptoms (13.3%)
Khan et al. (2017)	Sex Differences in Prodromal Symptoms in Acute Coronary Syndrome in Patients Aged 55 Years or Younger	Evaluate sex differences in prodromal symptoms including chest pain and non‐painful symptoms and health‐seeking behaviour in women and men aged 55 years or younger who had an ACS; Evaluate sex differences in pre‐hospital management of prodromal symptoms	MAPMISS	Canada, Switzerland and the United States	Prospective cross‐sectional study *N* = 1.145 patients (368 women)	Unusual fatigue (60%) Sleep disturbances (52.8%) Anxiety (44.4%) Palpitations (32.2%) Gastrointestinal symptoms (indigestion) (30%) Headache (24%) Cough (21.8%) Change in thinking and memory (21.8%) Dyspnea (20.8%) Loss of appetite (19.8%) Vision problems (14.7%) Difficulty breathing at night (13.5%)
Løvlien et al. (2006)	Are There Gender Differences Related to Symptoms of Acute Myocardial Infarction? A Norwegian Perspective	Compare the presentation of symptoms and disease behaviour among women and men with AMI in Nordic countries and evaluate various aspects that influence pre‐hospital delay	Self‐administered questionnaire	Norway	Cross‐sectional study *N* = 82 patients (38 woman)	Fatigue (52%) Dyspnea (26%) Chest pain (34%)
Løvlien et al. (2009)	Early Warning Signs of an Acute Myocardial Infarction and Their Influence on Symptoms During the Acute Phase With Comparisons by Gender	Investigate what types of prodromal symptoms occurred before AMI and how individuals responded to their prodromal symptoms with gender comparisons	Self‐administered questionnaire	Norway	Cross‐sectional study *N*= 533 patients (149 women)	Dyspnea (38%) Anxiety (35%) Nervousness (48%) Chest pain (44%) Fatigue (68%)
McSweeney et al. (2003)	Women's Early Warning Symptoms of Acute Myocardial Infarction	The most common prodromal symptoms of AMI how prodromal and acute symptoms relate to comorbidities and risk factors for coronary heart disease Whether prodromal symptoms were predictive of AMI symptomatology	MAPMISS	United States	Descriptive study *N*= 515 women	Unusual fatigue (70.7%) Sleep disturbances (47.8%) Dyspnea (42.1%) Gastrointestinal symptoms (indigestion) (39.4%) Anxiety (35.5%) Change in thinking and memory (23.9%)
McSweeney et al. (2010)	Racial Differences in Women's Prodromal and Acute Symptoms of Myocardial Infarction	Describe the prodromal and acute symptoms of AMI reported by women and determine if Hispanic and white women differ in symptoms Determine if the prodromal and acute symptoms reported by Hispanic and white women differ in severity and frequency when adjustments are made for known cardiovascular risk factors	MAPMISS	United States	Retrospective study *N* = 1270 women	Unusual fatigue (73%) Sleep disturbances (50.2%) Anxiety (45%) Dyspnea (45%) Gastrointestinal symptoms (frequent indigestion) (39%)
Joseph et al. (2021)	Atypical Manifestations of Women Presenting with Myocardial Infarction at Tertiary Health Care Center: An Analytical Study	Identify gender‐based differences in risk factors and clinical manifestations in patients presenting with AMI	Ad hoc questionnaire	India	Analytical cross‐sectional study *N*= 240 partecipants (120 women)	Dizziness, sweating, shortness of breath, vomiting, palpitations, fainting, back pain and fatigue (85%)
O'Keefe‐McCarthy et al. (2016)	Prodromal Symptoms Associated with Acute Coronary Syndrome Acute Symptom Presentation	Describe the prevalence, frequency and severity of prodromal symptoms in a sample of patients with ACS	PS‐SS scale—Pain Intensity—Speilberger State‐Trait Anxiety Personality Inventory	Canada	Descriptive cross‐sectional study *N*=121 participants (58 women)	Unusual pains (41.9%) Unusual fatigue (54.8%) Sleep disturbances (37.1%) Anxiety (61.3%) Dyspnoea (61.3%) Chest pain (67.7%) Headache (22.6%) Dizziness (35.5%) Others (54.8%)
Shi et al. (2020)	Sex Differences in Prodromal Symptoms and Individual Responses to Acute Coronary Syndrome	Examine potential sex differences in prodromal symptoms in Chinese patients with ACS and their individual responses to symptoms.	MAPMISS	China	Cross‐sectional study *N*= 806 patients (483 women)	Unusual fatigue (33.1%) Sleep disturbances (32.9%) Anxiety 26.2% Palpitations (20%) Dyspnea (30%) Gastrointestinal symptoms (indigestion) (46%)
Soltani et al. (2021)	The Association between Risk Factors and Prodromal Myocardial Infarction Symptoms: A Cross‐Sectional Study in Iran	Describe the prevalence and frequency of PS and risk factors in a sample of patients with AMI	MAPMISS	Iran	Cross‐sectional study *N*=154 patients (38 women)	Sleep disturbances and unusual fatigue (60%) Gastrointestinal symptoms (15.8%) Neurological symptoms (15.8%)

*Note:* ACS, acute coronary syndrome; AMI, acute myocardial infarction; MAPMISS, McSweeney Acute and Prodromal Myocardial Infarction Symptom Survey; MI, myocardial infarction; PS, prodromal symptoms; PS‐SS, Prodromal Symptoms‐Screening Scale.

### 4.3 Quality of the Studies

Quality assessment of the included studies was conducted using the QuADS tool (Harrison et al. 2021), as presented in Table 1. The studies included scored between 14 and 29 out of a maximum of 39 points. One of the studies scored less than 15 points. Ten studies achieved scores ranging from 16 to 29, and no study scored above 30. Studies assessed as ‘Serious’ using the ROBINS‐I tool (Sterne et al. 2016) (Table 2) were included with caution, while no study was deemed to have a ‘Critical’ risk. Among the 11 studies included, only three were rated as having ‘Moderate’ risk (Graham et al. 2008; Shi et al. 2020; Soltani et al. 2021) and eight as ‘Serious’ risk (McSweeney et al. 2003; Løvlien et al. 2006; Cole et al. 2012; O'Keefe‐McCarthy et al. 2016; Joseph et al. 2021). The main concerns leading to these ratings were in the domain of outcome measurement, due to the subjective nature of the measures (perceptions and self‐assessment), and participant selection due to the observational and retrospective design of most studies. Specifically, five studies exhibited a severe risk in the domain of ‘*bias due to confoundin*’ (McSweeney et al. 2003, 2010; Løvlien et al. 2009; O'Keefe‐McCarthy et al. 2016; Khan et al. 2017), six studies had a moderate risk (Graham et al. 2008; Cole et al. 2012; O'Keefe‐McCarthy et al. 2016; Khan et al. 2017; Shi et al. 2020; Soltani et al. 2021) and five studies showed a severe risk in the domain of ‘*bias due to selection of participants*’ (McSweeney et al. 2003, 2010; Løvlien et al. 2006 and 2009; Joseph et al. 2021). Only one study (Cole et al. 2012) demonstrated severe risk in the domain of ‘*bias in classification of interventions*’, unlike the moderate risk in the same domain reported in the studies by Khan et al. (2017) Feil (1937), O'Keefe‐McCarthy et al. (2016) and Joseph et al. (2021). The study by Joseph et al. (2021) alone showed severe risk in the domain of ‘*bias due to deviations from intended interventions*’; conversely, a moderate risk of ‘*bias due to missing data*’ was present in four studies (Løvlien et al. 2006; Graham et al. 2008; Khan et al. 2017; Shi et al. 2020). In the domain of ‘*bias in measurement of outcomes*', three studies (McSweeney et al. 2010; Cole et al. 2012; Joseph et al. 2021) presented severe risk and six studies (McSweeney et al. 2003; Løvlien et al. 2006 and 2009; Graham et al. 2008; Khan et al. 2017; Shi et al. 2020) a moderate risk; finally, only three studies (Cole et al. 2012; O'Keefe‐McCarthy et al. 2016; Khan et al. 2017) exhibited a moderate risk in the domain of ‘*bias in selection of the reported result*’.

### 4.4 Prodromal Symptoms

According to several studies, women report a notable prevalence and variety of prodromal symptoms of heart disease prior to acute events. Soltani et al. (2021) observed that 24% of women exhibit at least 2 prodromal symptoms, while 26% report at least 3, 10.5% at least 4 and 7.9% at least 5 symptoms. Khan et al. (2017) reported an average of 4.8 prodromal symptoms in women. Additionally, McSweeney et al. (2003) highlighted an average of 5.7 prodromal symptoms detected in women. Another study by McSweeney et al. (2010) on women from different ethnic groups revealed an average of 6 symptoms reported: 7.48 symptoms in black women, 6.98 in Hispanic women and 5.48 in white women (*p* < 0.01). 85% of women report prodromal symptoms, as reported by Khan et al. (2017), highlighting the significant prevalence of these symptoms in the female population (*p* < 0.0001). The literature shows that women report an average of six prodromal symptoms (Khan et al. 2017).

The period of presentation of prodromal symptoms, as reported in the various studies, varies widely and, in some cases, is not specified. For example, the study by Løvlien et al. (2009), which used a self‐administered questionnaire, considers symptoms that occurred in the year prior to AMI. In contrast, the study by Shi et al. (2020), which used the MAPMISS, looks at symptoms in the 6 months prior to the event. Other studies, such as those by Soltani et al. (2021) and Graham et al. (2008), while using the same tool (MAPMISS), observe a time frame of 3 months. In contrast, in some studies, such as those by McSweeney et al. (2003, 2010), Cole et al. (2012) and Khan et al. (2017), which also use MAPMISS, the period of symptom onset is not given. The studies by Løvlien et al. (2006) and Joseph et al. (2021), which are based on self‐administered questionnaires, also do not specify the onset of symptoms, as does the study by O'Keefe‐McCarthy et al. (2016), which used the PS‐SS instrument.

#### 4.4.1 Chest Pain and Other Types of Pain (Upper and Lower Limb Pain, Jaw/Teeth/Throat and Generalized Unusual Pain)

Khan et al. (2017) reports a percentage of 23.6% of chest pain, indicating it as a relevant symptom. Joseph et al. (2021) add further details, highlighting its location in the upper and interscapular chest part that radiates to the left arm (24.3%) of women, suggesting possible cardiac or musculoskeletal involvement. This data is also reported in the studies by Løvlien et al. (2006) and Løvlien et al. (2009) which highlight how this symptom is present respectively in 34% and 44% of cases, indicating its frequency and its importance in clinical evaluation. In both studies by McSweeney et al. (2003, 2010) an incidence of chest pain of 29.7% and 36%, pain, also defined with the term ‘discomfort’. If the women in the study by O'Keefe‐McCarthy et al. (2016) present this symptom for 67.7%, instead all the women enrolled in the study by Soltani et al. (2021) report chest pain or discomfort, underlining the wide range of conditions that can manifest with this symptom. In addition to chest pain, 66% of women report pain localized to the shoulder, 37% and 57% of back pain (Løvlien et al. 2006 and 2009). Data analysis shows that leg pain was studied in the article by Cole et al. (2012), which reported a significant association with an odds ratio of 2.10 (95% confidence interval: 1.29, 3.43). Arm pain is a symptom reported by women in 10 studies within the review: in the study by Cole et al. (2012) the odds ratio is 1.56 (95% CI: 1.14, 2.12) for pain in both arms. This data is confirmed in the studies by Løvlien et al. (2006) and from the study by Soltani et al. (2021) where the pain in both arms is reported from 23.7% to 61% of women. In this case, in addition to the term pain, women used terms like ‘discomfort, weak arms, heavy arms’ (Khan et al. 2017; McSweeney et al. 2003; Soltani et al. 2021) for 33.2%, 24.9% and, 1%, respectively. Other studies, however, report the presence of 45% of pain in one arm only (Løvlien et al. 2009), particularly the left arm (Joseph et al. 2021). Some studies also report the onset of pain in the jaw/teeth/throat in 6% (Khan et al. 2017) and 40% (Løvlien et al. 2006). Unusual, generalized pain is reported by O'Keefe‐McCarthy et al. (2016) in 41.9% and by Graham et al. (2008) in 54.5%.

#### 4.4.2 Fatigue

Fatigue is a symptom present in eleven studies included in the review. From the data provided, we can observe that fatigue is reported with prevalence rates ranging from a minimum of 33.1% to a maximum of 85%. Specifically, Løvlien et al. (2006 and 2009) highlighted a high prevalence of fatigue, with percentages of 52% and 68%, respectively. McSweeney et al. (2003, 2010) reported even higher significant values, with percentages reaching 70.7% and 73%. Similarly, both Khan et al. (2017) and Soltani et al. (2021) detected a 60% rate of subjects reporting fatigue as a prodromal symptom. Other authors, such as Cole et al. (2012), Graham et al. (2008), O'Keefe‐McCarthy et al. (2016) and Shi et al. (2020) also report unusual fatigue of 2.16 (odds ratio, with a 95% confidence interval ranging from 1.62 to 2.89), 52.4%, 54.8% and 33.1%, respectively. Notably, an observation by Joseph et al. (2021) recorded a high prevalence of 85% of subjects reporting fatigue as a prodromal symptom. This particularly high figure underscores the importance of fatigue as an early signal of various diseases.

#### 4.4.3 Anxiety

In the eight studies included in the review, anxiety emerges as a significant and common symptom. For instance, the study conducted by Cole et al. (2012) highlighted an odds ratio for anxiety of 2.21, with a 95% confidence interval ranging from 1.71 to 2.84. Graham et al. (2008) reported that anxiety, along with fatigue, was identified in 52.4% of cases, indicating that anxiety might be a relevant component of prodromal symptoms. Khan et al. (2017) reported a percentage of 44.4% of patients who reported anxiety, further confirming its significance. Løvlien et al. (2009) and McSweeney et al. (2003, 2010) reported a prevalence of 35% and 45%, while O'Keefe‐McCarthy et al. (2016) reported a higher percentage of 61.3%. Shi et al. (2020) report a lower level of anxiety (26.2%) compared to that reported in other studies. Overall, we can see that anxiety is a common symptom reported in many of the studies included, with prevalence percentages varying between 26.2% and 61.3%.

#### 4.4.4 Sleep Disorders

Khan et al. (2017) reported that more than half of the participants, specifically 52.8%, reported sleep disturbances as a prodromal symptom. McSweeney et al. (2003, 2010) also highlighted a significant presence of sleep disorders, with percentages of 47.8% and 50.2% respectively, among the women involved in their studies. O'Keefe‐McCarthy et al. (2016) confirmed the importance of this symptom, finding that 37.1% of subjects experienced sleep disturbances during the prodromal period. Shi et al. (2020) reported a percentage of 32.9% of participants who reported sleep disturbances as a prodromal symptom. Finally, Soltani et al. (2021) noted that 60% of subjects reported sleep disturbances along with fatigue as prodromal symptoms. These data indicate that sleep disturbances are a relevant and widespread symptom in the early stages of some pathological conditions, with a frequency varying between 32.9% and 52.8%.

#### 4.4.5 Dyspnea and Respiratory Problems

The studies analyzed highlighted the significant presence of dyspnoea as a prodromal symptom of AMI, with a frequency ranging from 20.8% to 61.3%. Khan et al. (2017) report a prevalence percentage of dyspnea of 20.8%; Løvlien et al. (2006) at 26% and subsequently in a 2009 study (Løvlien et al. 2009) at 38%; Shi et al. (2020) at 30%; McSweeney et al. (2003) and McSweeney et al. (2010) reported frequencies of 42.1% and 45% among their participants, reaching up to 61.3% in the study by O'Keefe‐McCarthy et al. (2016). Joseph et al. (2021) also report the presence of this symptom in their study. Khan et al. (2017) further document the presence of other respiratory problems, such as a frequency of 21.8% for coughing and 13.5% for night time breathing difficulty.

#### 4.4.6 Changes in Thinking and Memory

The studies by Cole et al. (2012), Khan et al. (2017) and McSweeney et al. (2003) discuss changes in memory and thinking during the prodromal period. According to Cole et al. (2012), the change in thinking and memory has an odds ratio of 1.47 (95% CI: 1.12–1.94), Khan et al. 2017 at 21.8%, while McSweeney et al., in 2003, report that the change in thinking and memory was observed in 23.9% of cases.

#### 4.4.7 Gastrointestinal Symptoms

Cole et al. (2012) report an odds ratio of 1.29 (95% CI: 1.00–1.67) for gastrointestinal symptoms. Graham et al. (2008) indicate a frequency of 3.4% for such symptoms. Khan et al. (2013), Shi et al. (2020) and Soltani et al. (2021) respectively 30%, 46% and 15.8% including indigestion. Also, in studies of McSweeney et al. (2003, 2010) report frequencies for indigestion symptom of 39.4% and 39% are reported, values also confirmed by Joseph et al. (2021) that reporting vomiting as a symptom. Gastrointestinal symptoms, reported in the review studies, thus have a frequency ranging between 3.4% and 39%.

#### 4.4.8 Headache

Headache is a relevant symptom that can be associated with and predict acute myocardial infarction, and its presence can vary between 15.8% and 24% in women with AMI. Specifically, in a study conducted by Cole et al. (2012), the headache presents an odds ratio of 1.54 (95% CI: 1.03–2.29). Graham et al. (2008) identified headache as prodromal symptoms present in 15.8% of women, associating it with anxiety and fatigue. Khan et al. (2017) reported that headache was observed in 24% of cases, data comparable to the study conducted by O'Keefe‐McCarthy et al. (2016); the percentage of patients with headaches among them is 22.6%.

#### 4.4.9 Other Prodromal Symptoms

In addition to the symptoms discussed previously, there are other prodromal symptoms of acute myocardial infarction in women not to be underestimated. In the study by Løvlien et al. (2009), women were significantly more likely than men to report being nervous (48%) (*p* < 0.01), to present palpitations, investigated in the study by Khan et al. (2017) and Shi et al. (2020), respectively with percentages of 32.3% and 20%. Women also may report loss of appetite (19.8%), vision problems (14.7%) and dizziness, as reported in the study by Joseph et al. (2021) (85%) or with a percentage of 35.5% in the study by O'Keefe‐McCarthy et al. (2016).

## 5 Discussion

This systematic review provides a synthesis and quantitative analysis of the evidence available in the literature on prodromal symptoms in women before SCA. The results confirm that women experience a wide range of prodromal symptoms, with prevalence rates as high as 85% (McSweeney et al. 2003, 2010; Khan et al. 2017). The review points out that women are less likely to manifest chest pain traditionally associated with AMI (Chandrasekhar et al. 2018; Blakeman et al. 2020), presenting instead with more heterogeneous symptoms (McSweeney et al. 2003, 2010; Khan et al. 2017; Blakeman and Booker 2016). This has relevant diagnostic implications, as chest pain, while the most commonly known symptom, is not always a primary indicator in women. Indeed, chest pain associated with AMI is a particularly relevant in females, as women tend to have worse outcomes even with timely diagnosis and emergency treatment (DeVon et al. 2008). Although chest pain remains a significant symptom of AMI, it is not always the most common prodromal symptom in women, who may experience a wider range of early indicators (Albarran et al. 2007; DeVon et al. 2008; Milner et al. 2004).

In fact, some studies included in this review indicate that many women do not frequently report prodromal chest pain, and although present it is described as a sensation of pressure, constriction or discomfort (Albarran et al. 2007; Asghari et al. 2022). This aspect suggests the urgency of revising traditional diagnostic paradigms and adapting clinical guidelines to better address the gender‐specific manifestations of AMI (Albarran et al. 2007). Despite these differences, the 2022 AHA/ACC guidelines (Ramirez et al. 2022) point out that men can also manifest similar symptoms, such as pressure sensations or prodromal chest discomfort, thus reducing the need for exclusively gender‐based assessment (Gimenez et al. 2014). Consequently, it is essential to overcome the idea that AMI is always exclusively associated with chest pain, regardless of gender, as symptoms can vary significantly. The differences in symptoms between men and women with SCA can be explained by the way chest pain is transmitted through activation of the sympathetic and parasympathetic nerves. The afferent fibres of the sympathetic nerves are more concentrated along the left anterior coronary artery, whereas the parasympathetic fibres predominate along the right coronary artery and circumflex branch. Consequently, the area affected during an acute myocardial infarction may influence the predominance of sympathetic or vagal nerve activation. In particular, parasympathetic nervous system activation tends to be more common in patients with a right‐sided dominated coronary system, a feature found more frequently in women. This mechanism could help explain gender differences in the symptomatic presentation of AMI (Khan et al. 2013; Arora and Bittner 2015; Safdar et al. 2014; Canto 2007). The three most common prodromal symptoms in women reported in the included studies are fatigue, anxiety and sleep disturbance. Fatigue, in particular, is described as unexplained and so intense that it prevents the performance of normal daily activities, such as making the bed (McSweeney et al. 2010; Albarran et al. 2007) and having a lack of energy to complete daily tasks (Bowles 2013; Bruno 2013). This symptom, reported to have a prevalence of up to 70% (Albarran et al. 2007; Lindgren et al. 2008; Berg et al. 2009), was identified as the most common in seven out of nine studies in the review by Blakeman and Booker (2016). However, fatigue is a complex phenomenon (Whitehead 2009), impacting not only physical health but also cognitive processes, referred to as ‘mental fog’ (Ream and Richardson 1997). The complexity of this phenomenon may be one of the main reasons for the large variation in symptom prevalence, ranging from 33.1% to 85% in the included studies, along with other factors. This variability can be explained by differences in the characteristics of the female population analyzed (e.g., age and comorbidities), variations in the methods used to assess symptoms (such as the use of self‐completed questionnaires versus clinical assessments), and the adoption of different criteria or definitions to identify symptoms. In addition, cultural, regional and health system factors may influence how symptoms are reported or recognized.

Anxiety reported by many women in the weeks before the event represents a significant prodromal symptom (Miller et al. 2009; Smeijers et al. 2016). The scientific literature defines this condition as anticipatory anxiety, which is not only associated with an increased risk of AMI but also contributes to a worse prognosis and exacerbates the severity of myocardial ischemia in the female sex (Bekendam et al. 2021), due to the pathophysiological mechanisms it triggers (Celano et al. 2015). Specifically, anxiety reduces parasympathetic nervous system activity and increases heart rate, thus leading to increased oxygen consumption by the heart. This can promote the onset of myocardial ischemia, even in the absence of severe coronary artery disease (Chalmers et al. 2014; Hofmann et al. 2014).

Sleep disturbances have also been associated with an increased risk of cardiovascular disease in women, a risk that increases in women who sleep < 6 h per night (Chandola et al. 2010). Specifically, according to the study by Meisinger et al. (2007), women who sleep 5 h a night, compared to women who sleep 8 h, have a higher incidence of AMI; as well as reduced sleep duration is associated with elevated levels of inflammatory markers such as interleukin‐6 and C‐reactive protein, which contribute to cardiovascular risk (Miller et al. 2009).

In addition to the three most frequently reported prodromal symptoms, women also report other less frequent signs, including gastrointestinal complaints, headache, cognitive changes, breathing difficulties, nervousness, palpitations, loss of appetite and pain. Although the incidence of these symptoms varies among studies, they are often overlooked or attributed to unrelated causes by the patients themselves (Lichtman et al. 2015; Soltani et al. 2021). Nevertheless, their presence should be carefully evaluated as a possible indicator of an impending cardiac event.

The number and severity of prodromal symptoms is another aspect that comes from this review. Indeed, it was found that women report on average about six symptoms before an acute event, a finding that significantly affects their pre‐event health status. Specifically, women tend to have at least two of the three ‘most common’ prodromal symptoms, such as fatigue and sleep disturbance, with similar but severe intensity; as for the other ‘less common’ symptoms, such as shortness of breath and indigestion, these are classified as severe, moderate or mild with the same frequency (McSweeney et al. 2003, 2010).

The period of presentation of prodromal symptoms reported in the various studies shows considerable methodological heterogeneity, reflecting the breadth and complexity of research in this field. Studies such as that of Løvlien et al. (2009), which consider the period of 1 year, provide a broad perspective useful for identifying prodromal symptoms that may vary over time; similarly, the approach of Shi et al. (2020), which focuses on the preceding 6 months, and that of Soltani et al. (2021) and Graham et al. (2008), which further reduce the period to 3 months, may provide greater temporal precision. The absence of a defined time window in some studies, such as those by McSweeney et al. (2003, 2010), Cole et al. (2012) and Khan et al. (2017), limits the ability to compare results across research and complicates the synthesis of generalisable conclusions. The same is true for studies using self‐completed questionnaires, such as those in Løvlien et al. (2006) and Joseph et al. (2021), or alternative instruments such as the PS‐SS in O'Keefe‐McCarthy et al. (2016), which do not provide a clear temporal definition.

Early recognition of symptoms is crucial to reducing the risk of adverse cardiac events. However, for example, despite fatigue being an important symptom of AMI in women (Blakeman and Stapleton 2018; Blakeman et al. 2020), only 10% of women recognize fatigue as such (Mosca et al. 2013). This highlights the need for more targeted education for patients, as well as upgrading the skills of healthcare providers, particularly nurses, who are uniquely positioned to identify prodromal symptoms and promote a timely response (Giordano et al. 2024; Siebens et al. 2007). Studies such as that of Birnbach et al. (2020) show that advanced nursing education can improve the ability to recognize premonitory symptoms, increasing clinical response and reducing delays in treatment.

In summary, this systematic review highlights the critical need for increased awareness of prodromal symptoms of ACS in women. By integrating these findings into clinical practice, health care providers can improve the accuracy and timeliness of diagnosis, ultimately reducing morbidity and mortality. Future research should aim to standardize methodologies for symptom assessment and explore targeted interventions to improve early recognition and management of cardiovascular risk in women. Addressing these gaps will be critical to achieving equitable and effective care for this population.

This review has some limitations. First, the retrospective nature and reliance on convenience sampling in many of the included studies may introduce allocation bias. Such methodologies compromise the generalisability of the results and may bias outcomes toward specific patient demographic groups. Another limitation is the inclusion criterion restricted to women with confirmed diagnoses of ACS. This excludes a significant portion of the female population who may experience similar prodromal symptoms without a subsequent diagnosis, limiting understanding of the prevalence of these symptoms among the undiagnosed. Women may not remember their symptoms accurately or may unintentionally bias their responses, which may affect the reliability of the data. In addition, reliance on self‐reported rather than standardized instruments or the use of assessment criteria by health professionals could lead to underestimation of some symptoms.

These instruments generally include a predefined list of symptoms, limiting participants to report only those symptoms included or to provide detailed descriptions outside of those given in the questionnaires. A significant limitation of the studies included in this systematic review concerns the considerable variability and, in some cases, lack of specification of the period of presentation of prodromal symptoms. Some studies consider large time intervals, such as a full year before the acute ischemic event, while others limit observation to 6 or 3 months, potentially neglecting longer periods of symptomatology. This is particularly relevant considering that qualitative studies in the literature indicate that prodromal symptoms in women can last an average of 4 to 6 months (Rosenfeld et al. 2005; Coventry et al. 2017; Arslanian‐Engoren and Scott 2017). Moreover, in some cases, the period of onset is not specified at all in the review, leaving an important gap in understanding the chronology of symptoms. This methodological heterogeneity not only makes it difficult to directly compare the results of the various studies but also complicates the identification of consistent patterns in prodromal symptoms, thereby limiting opportunities to develop robust evidence‐based clinical guidelines.

### 5.1 Limitations

The review presented significant methodological heterogeneity among the included studies, with variability in study designs (cross‐sectional, retrospective and descriptive), measurement instruments (e.g., MAPMISS, self‐administered questionnaires, PS‐SS) and characteristics of the analyzed populations. This diversity made it impossible to conduct a meta‐analysis, limiting the synthesis of results to a narrative analysis and reducing the generalizability of conclusions. In addition, the predominant use of self‐reported data introduced potential recall bias and a possible underestimation or overestimation of some symptoms. Another limitation concerns the difficulty in distinguishing data on female‐specific premonitory symptoms of myocardial infarction in studies with a male comparison group.

Despite these limitations, the review methodology—being a systematic review—is a major strength, ensuring a structured and rigorous approach to data collection and analysis. This review provides an exhaustive overview focused solely on the prodromal symptoms of acute myocardial infarction in women, addressing an important gap in the literature. Moreover, the inclusion of studies not previously considered in other reviews adds new evidence and perspectives to the existing knowledge base. This expansion of research scope enhances our understanding of the prodromal symptoms associated with acute myocardial infarction in women, offering a more comprehensive and updated synthesis of the available evidence. Ultimately, the results, although interesting, must be interpreted with relative caution in light of the limitations.

### 5.2 Implications and Recommendations for Practice

Recognizing prodromal symptoms can significantly aid in the early identification of women at risk for coronary heart disease, allowing for a tailored approach to managing cardiovascular risk factors and reducing delays in seeking treatment during an acute coronary event. However, long‐term prospective studies are essential to fully understand the clinical significance of prodromal symptoms, especially in women with cardiac diseases. Further research is needed to confirm whether prodromal symptoms can predict future adverse cardiac events, as suggested in the study protocol by Giordano et al. (2024), and if they are more predictive than common comorbidities, as the current literature on this relationship is either limited or has certain limitations. It is also crucial to examine the effectiveness of prodromal scores in predicting abnormal outcomes in cardiovascular diagnostic tests and to assess whether there are differences in symptom manifestations exist between women from different ethnic groups. Similarly, it is necessary to examine how men may experience different prodromal symptoms than women, not only in terms of the type of symptoms reported, but also in terms of their frequency and intensity. These aspects may suggest the need for individualized diagnostic and therapeutic approaches that are more targeted to the specificities of each gender and ethnic group.

In addition, the development of a recognized nomenclature of prodromal symptoms could improve our understanding of the epidemiology of the prevalence and incidence of prodromal symptoms of myocardial infarction in women and determine how these symptoms may vary based on ethnicity and risk factor profiles. Additionally, experts in the symptomatology of myocardial infarction in women should collaborate to validate and select a uniform tool for predicting infarction symptoms of myocardial infarction, thereby enabling the use of a predictive scale in clinical practice.

Educating women about prodromal symptoms and involving caregivers in their identification may facilitate a timely initiation of cardiac evalutation. Early recognition of prodromal symptoms can not only identify individuals at risk for developing coronary disease, but may also reduce morbidity and mortality associated with coronary disease in women.

## 6 Conclusions

This systematic review provides an understanding of the prodromal symptoms associated with ACS in women. The results show that women may experience more prodromal symptoms during the ACS, such as fatigue, anxiety and sleep disorders. Although these symptoms are common, they are often unrecognized or misattributed to non‐cardiac causes, leading to delays in diagnosis and treatment. However, it is important to note that there is still no definitive understanding of the differences in prodromal symptoms between men and women and that the complexity of these symptoms cannot be stated with certainty due to the nature of the included studies.

## Author Contributions

Conceptualization: V.G. and A.G. Methodology: V.G., A.G., and R.N. Software: V.G., A.G., and R.N. Validation: V.G., A.G., and R.N. Formal analysis: V.G. and A.G. Investigation: V.G., A.G., and A.G. Resources: V.G., A.G., and T.R. Data curation: V.G., A.G., and C.M. Writing – original draft preparation: V.G., A.G., and C.M. Writing – review and editing: V.G., A.G., R.N., and T.R. Visualisation: V.G. and A.G. Supervision: V.G. and T.R. Project administration: V.G. and A.G. All authors have read and agreed to the published version of the manuscript.

## Ethics Statement

The authors have nothing to report.

## Conflicts of Interest

The authors declare no conflicts of interest.

## Supporting information


Appendix S1.

## Data Availability

The data that support the findings of this study are available from the corresponding author upon reasonable request.
