# Supplementary material for: Prodromal Symptoms of Acute Myocardial Infarction in Women: A Systematic Review of Current Evidence
Source: Nurs Open. 2025 Apr 22;12(4):e70211. doi: 10.1002/nop2.70211 (PMC12012308; doi:10.1002/nop2.70211)
Supplement: Supplementary file 1 — Appendix S1. [file NOP2-12-e70211-s001.docx]

**Supplementary**

**SEARCH STRINGS**

| **CINAHL** | ( woman or women or female or females ) AND (prodromal symptoms OR premonitory symptoms) AND ( acute coronary syndrome AND acute myocardial infarction or ami or heart attack or infarction, acute myocardial or mi or myocardial infarction or myocardial infarction acute or acute disease or myocardial infarction) |
| --- | --- |
| **PUBMED** | **(woman OR women OR gender OR female OR sex) AND ("Myocardial Infarction" OR "acute Myocardial Infarction" OR "Heart Attack" OR "ischemic heart disease" OR "acute coronary syndrome") AND ("prodromal symptoms" OR "early warning")**  ("womans"[All Fields] OR "women"[MeSH Terms] OR "women"[All Fields] OR "woman"[All Fields] OR "women s"[All Fields] OR "womens"[All Fields] OR ("womans"[All Fields] OR "women"[MeSH Terms] OR "women"[All Fields] OR "woman"[All Fields] OR "women s"[All Fields] OR "womens"[All Fields]) OR ("gender identity"[MeSH Terms] OR ("gender"[All Fields] AND "identity"[All Fields]) OR "gender identity"[All Fields] OR "gendered"[All Fields] OR "gender s"[All Fields] OR "gendering"[All Fields] OR "genderized"[All Fields] OR "genders"[All Fields] OR "sex"[MeSH Terms] OR "sex"[All Fields] OR "gender"[All Fields]) OR ("femal"[All Fields] OR "female"[MeSH Terms] OR "female"[All Fields] OR "females"[All Fields] OR "female s"[All Fields] OR "femals"[All Fields]) OR ("sex"[MeSH Terms] OR "sex"[All Fields])) AND ("Myocardial Infarction"[All Fields] OR "acute Myocardial Infarction"[All Fields] OR "Heart Attack"[All Fields] OR "ischemic heart disease"[All Fields] OR "acute coronary syndrome"[All Fields]) AND ("prodromal symptoms"[All Fields] OR "early warning"[All Fields]) |
| **APA PsycArticles, APA PsycInfo** | (woman or women or female or females ) AND (early warning OR prodromal symptoms OR premonitory symptoms) AND ( acute coronary syndrome OR acute myocardial infarction or ami or heart attack or infarction, acute myocardial or mi or myocardial infarction or myocardial infarction, acute or acute disease or myocardial infarction) |
| **EMBASE** | ('female'/exp OR female) AND ('ischemic heart disease'/exp OR 'ischemic heart disease' OR 'acute coronary syndrome') AND ('prodromal symptom'/exp OR 'prodromal symptom') |
